# The COVID-19 Diagnostic Technology Landscape: Efficient Data Sharing Drives Diagnostic Development

**DOI:** 10.3389/fpubh.2020.00309

**Published:** 2020-06-18

**Authors:** Eric R. G. R. Aguiar, Jesús Navas, Luis G. C. Pacheco

**Affiliations:** ^1^Department of Biological Sciences, Center of Biotechnology and Genetics, State University of Santa Cruz (UESC), Ilhéus, Brazil; ^2^Department of Molecular Biology, Universidad de Cantabria, Santander, Spain; ^3^Department of Biotechnology, Institute of Health Sciences, Federal University of Bahia (UFBA), Salvador, Brazil

**Keywords:** COVID-19, diagnostics, Point-of-care (POC), data sharing, molecular diagnosis, immunoassay

## Introduction

Since the first case descriptions in December 2019, the COVID-19 pandemic has prompted the development of diagnostic technologies at an unprecedented pace, and the pattern of collaborative scientific data sharing during this period has followed a similar path. A recent bibliometric study demonstrated that the research publication response to the COVID-19 pandemic was much more effective than in other recent epidemic events, namely the 2015–16 Zika virus epidemic and the 2014–16 Ebola virus outbreak in West Africa (1). Concerning only preprint publications, there were over 2,500 articles related to COVID-19 in the first 4 months of the pandemic, as opposed to only 88 articles in total related to both the Zika and Ebola viruses in the same epidemiological periods. Additionally, by the end of April, the total number of COVID-19 publications, including preprints and peer-reviewed articles, had already surpassed 16,000 (1). When we searched PubMed specifically for scientific publications related to COVID-19 diagnostics (search terms: COVID-19 AND Diagnostics), it returned at least 930 specific papers in the first 5 months of the COVID-19 epidemic period (limited to December 2019–April 2020), while a similar search for Zika virus retrieved only nine publications related to diagnostics in the same time period (limited to March 2015–July 2015). Other recent publications have also discussed the efficiency of open data sharing during the early part of the COVID-19 pandemic, particularly of epidemiological and diagnostic data, and how it contributed to early interventions (2, 3).

**Figure 1:**
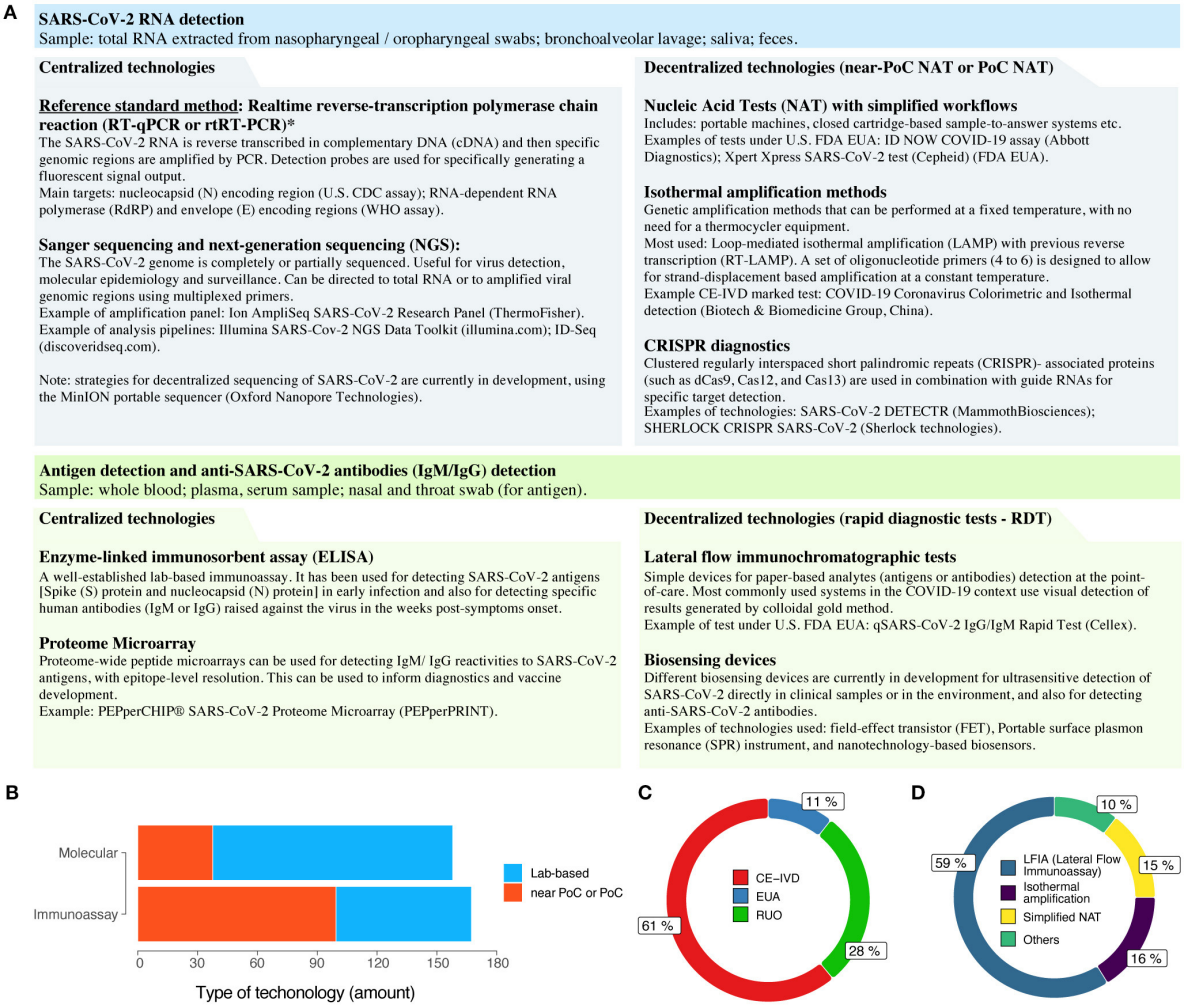
The COVID-19 diagnostic technology landscape. **(A)** A (non-exhaustive) list of the current and emerging technologies for laboratory-based or decentralized (near or at the point-of-care) COVID-19 diagnosis. Methods for clinical diagnosis of COVID-19, such as chest computed tomography, are discussed elsewhere (6, 7, 11). *Disambiguation: despite being frequently used in the COVID-19 context, the abbreviation RT-PCR is more appropriate to the traditional method of reverse-transcription PCR. For real-time (quantitative) reverse-transcription PCR, such as in SARS-CoV-2 detection, it is more appropriate to use RT-qPCR or rtRT-PCR. FDA EUA, US Food and Drug Administration Emergency Use Authorization (EUA); PoC, point-of-care; NAT, nucleic acid test. **(B)** Categories of commercially manufactured COVID-19 diagnostic tests, as of late Apr 2020. **(C)** Regulatory status of the available tests. EUA, Emergency Use Authorization; CE-IVD, Conformité Européenne (EU certification)-*in vitro* diagnostics; RUO, research use only. **(D)** Major technologies used in current point-of-care diagnostic tests for COVID-19.

The speed by which viral genomic sequences were made publicly available during the COVID-19 pandemic also demonstrates the fast pace of data sharing during the period. As early as December 31, 2019, 19 genomic sequences of the SARS-CoV-2 virus were already available through the GISAID database (gisaid.org), which now has over 40,000 viral genome sequences shared by laboratories around the globe. As a comparison, during the Ebola virus outbreak, it took nearly 3 years for the number of sequenced viral genomes to reach 1,500 sequences (4). The early availability of SARS-CoV-2 genomic sequences contributed to the rapid development of the gold standard molecular diagnostic assays for COVID-19, based on reverse-transcription polymerase chain reaction (RT-qPCR), made available by the World Health Organization (WHO) and the US Centers for Disease Control and Prevention (CDC), still in early 2020 (5–7). Additionally, it also contributed to the development of streamlined protocols for complete viral genome sequencing and analysis (8, 9) and of lab-based serology assays that use recombinantly-produced SARS-CoV-2 proteins (10) (Figure 1A).

## Rapid Data Sharing Contributed To Development and Validation of Covid-19 Diagnostics

A good example demonstrating how rapid data sharing contributed to the development of diagnostics during the COVID-19 pandemic is shown by the first RT-qPCR assay design developed by researchers from the Charité – Universitätsmedizin Berlin Institute of Virology in Germany (12). The first SARS-CoV-2 viral genome sequence was made publicly available for immediate public health support as soon as 10 days after official reporting of the early cases of atypical pneumonia in China to the WHO. Only 3 days later (on January 13, 2020), the first RT-qPCR assay was made available to the international community. A few days later, positive controls were already available through the European Virus Archive (EVAg) repository (13). Soon after, on February 4, 2020, the US Food and Drug Administration (FDA) issued an emergency use authorization (EUA) to the CDC's 2019 Novel Coronavirus (2019-nCoV) Real-Time RT-PCR Diagnostic Panel. It did not take long for new studies describing SARS-CoV-2 viral load kinetics in different samples to be published for the different genomic targets (N, E, and RdRP), and this contributed to improvements in diagnostic protocols early in the epidemic. Compared to Zika virus epidemic, it was only nearly 1 year after first case descriptions in Brazil that the US FDA issued an EUA for the Trioplex assay on March 17, 2016.

The widespread adoption of preprint servers (such as medrxiv.org and biorxiv.org) for sharing research data before peer review has also allowed rapid publication of studies evaluating the performances of different diagnostic technologies and has contributed to a clearer understanding of emerging technologies that will potentially aid in the diagnosis and surveillance of COVID-19 in the near future (Figure 1A). Different studies have demonstrated that preprint publications were underutilized during the Zika and Ebola virus epidemics, despite being important tools for accelerating scientific development during disease outbreaks (1, 14). Now, COVID-19 related preprint publications have permitted foreseeing emerging roles for technologies based on loop-mediated isothermal amplification (LAMP) and CRISPR-based diagnostics, as these technologies are indeed appearing now in peer-reviewed publications and starting to reach commercial applications. In an interesting recent development, for example, isothermal amplification by reverse-transcription (RT)-LAMP was combined with specific CRISPR/Cas12 detection of SARS-CoV-2 amplified targets and with visual readout by lateral flow assay (15).

## The Covid-19 Point-of-Care Diagnostic Technology Landscape

Now, we can easily follow the development of new COVID-19 diagnostic technology into commercial products due to data sharing initiatives, such as the Foundation for Innovative New Diagnostics (FIND; finddx.org/covid-19/) and 360 Dx coronavirus test tracker (360dx.com). From data made available on over 590 COVID-19 diagnostic tests (as of April 24, 2020), we can have a clear view of the point-of-care (PoC) diagnostic technology landscape (Figure 1B). Although the numbers of commercially manufactured COVID-19 molecular tests and immunoassays are similar, there is clearly a higher proportion of decentralized tests that are based on immunoassays when compared to molecular methods (Figure 1B). This is probably due to the technological maturity of colloidal-gold immunochromatographic assays. Conversely, the greater number of lab-based commercial molecular tests for COVID-19 is due to the high number of companies offering RT-qPCR based kits (Figure 1B). Regarding regulatory status, there is a high proportion of CE-marked PoC tests that comply with the relevant European Union regulations (Directive 98/79/EC on *in vitro* diagnostics), although this does not necessarily mean that these tests are commercially available in Europe (Figure 1C). Additionally, novel EUAs for COVID-19 tests are being granted in a continuous basis by regulatory agencies worldwide, including the US FDA (fda.gov) and the Brazilian ANVISA (http://portal.anvisa.gov.br/coronavirus).

As of late April 2020, the WHO still did not recommend the use of PoC rapid immunodiagnostic tests (RDTs) for patient care and public health decision-making in the COVID-19 context (16, 17). However, most of the commercially available tests to date are in fact based on lateral flow immunoassay (LFIA) technologies for detecting SARS-CoV-2 antigens or human IgM/IgG antibodies (Figure 1D). Regarding PoC or near-PoC commercially manufactured molecular tests, two technological strategies are clear: (i) methods that provide simplified workflows for nucleic acid amplification tests (NAATs); and (ii) methods based on isothermal amplification by LAMP. In the former category, tried-and-tested diagnostic platforms with simplified sample-to-results workflows have already been introduced by major companies, such as the Xpert Xpress SARS-CoV-2 test (Cepheid) and the ID Now COVID-19 assay (Abbott Diagnostics).

## Conclusion

The rapid development of diagnostic technology is an essential component of an epidemic preparedness strategic plan (18). Accordingly, the technological landscape of the development of COVID-19 diagnostics is rapidly evolving, with new information being generated on a daily basis. Different platforms for open and fast data sharing have been contributing to this rapid diagnostic development, that include: fast availability of genomic data in public sequence repositories (e.g., gisaid.org); open collaboration in preliminary data analysis using science community blogs and discussion forums (e.g., virological.org); publication of periodic reports by universities and international organizations (e.g., the WHO); real-time sharing of diagnostic validation results (e.g., finddx.org); and particularly the use of preprint servers for early publication of research studies (e.g., medRxiv and bioRxiv). Recent studies have shown that these fast publication platforms are driving much of the debate about the COVID-19 pandemic, despite the intrinsic limitations associated with the unregulated sharing of research results at such fast pace (19). Therefore, to ensure the integrity and quality of rapidly shared studies, the research community is already putting into practice several control mechanisms, at various levels (20–22). From researcher-led initiatives, that include the creation of open peer-review platforms for improving the quality of COVID-19-related preprints (21), to publisher-led initiatives, such as the fast peer-review of research studies previously posted to non-peer reviewed platforms, these mechanisms will altogether contribute to guarantee the credibility of speedy information delivery during the pandemic (19–22).

This opinion paper was not meant to present exhaustive information on COVID-19 diagnosis, but rather to make an overview of currently available technologies in the academic and commercial settings for laboratory and PoC testing. For excellent reviews on strategies for COVID-19 diagnosis, we refer the readers to Cheng et al. (6), Tang et al. (7), and Udugama et al. (11). Besides, up-to-date information on COVID-19 diagnostic technology can be found at the following sources:

- WHO Coronavirus disease (COVID-19) technical guidance: Laboratory testing for 2019-nCoV in humans (23)
- FIND: COVID-19 Diagnostics Resource Center (24)
- U.S. FDA Coronavirus Disease 2019 (COVID-19) (25).

As new COVID-19 diagnostic technologies are introduced, studies aimed at validating their usefulness in clinical settings will be of crucial importance (26). In this sense, collaborative data sharing on SARS-CoV-2 diagnostic performance evaluation, such as the initiatives led by FIND (24) and the WHO, will contribute to rapid adoption of new diagnostic technology and will inform public health decisions on a global scale.

## Author Contributions

EA, JN, and LP conceived the study. EA and LP collected data and prepared data presentation. All authors wrote, reviewed, and approved the final version of the manuscript.

## Conflict of Interest

The authors declare that the research was conducted in the absence of any commercial or financial relationships that could be construed as a potential conflict of interest.
